# Dependence on the MUC1-C Oncoprotein in Classic, Variant, and Non–neuroendocrine Small Cell Lung Cancer

**DOI:** 10.1158/1541-7786.MCR-22-0165

**Published:** 2022-05-25

**Authors:** Atsushi Fushimi, Yoshihiro Morimoto, Satoshi Ishikawa, Nami Yamashita, Atrayee Bhattacharya, Tatsuaki Daimon, Hasan Rajabi, Caining Jin, Masayuki Hagiwara, Yota Yasumizu, Zhou Luan, Wenhao Suo, Kwok-Kin Wong, Henry Withers, Song Liu, Mark D. Long, Donald Kufe

**Affiliations:** 1 Dana-Farber Cancer Institute, Harvard Medical School, Boston, Massachusetts.; 2 Laura and Isaac Perlmutter Cancer Center, New York University Langone Medical Center, New York, New York.; 3 Department of Biostatistics & Bioinformatics, Roswell Park Comprehensive Cancer Center, Buffalo, New York.

## Abstract

Small cell lung cancer (SCLC) is a recalcitrant malignancy defined by subtypes on the basis of differential expression of the ASCL1, NEUROD1, and POU2F3 transcription factors. The MUC1-C protein is activated in pulmonary epithelial cells by exposure to environmental carcinogens and promotes oncogenesis; however, there is no known association between MUC1-C and SCLC. We report that MUC1-C is expressed in classic neuroendocrine (NE) SCLC-A, variant NE SCLC-N and non-NE SCLC-P cells and activates the MYC pathway in these subtypes. In SCLC cells characterized by NE differentiation and DNA replication stress, we show that MUC1-C activates the MYC pathway in association with induction of E2F target genes and dysregulation of mitotic progression. Our studies further demonstrate that the MUC1-C→MYC pathway is necessary for induction of (i) NOTCH2, a marker of pulmonary NE stem cells that are the proposed cell of SCLC origin, and (ii) ASCL1 and NEUROD1. We also show that the MUC1-C→MYC→NOTCH2 network is necessary for self-renewal capacity and tumorigenicity of NE and non-NE SCLC cells. Analyses of datasets from SCLC tumors confirmed that MUC1 expression in single SCLC cells significantly associates with activation of the MYC pathway. These findings demonstrate that SCLC cells are addicted to MUC1-C and identify a potential new target for SCLC treatment.

**Implications:**

This work uncovers addiction of SCLC cells to MUC1-C, which is a druggable target that could provide new opportunities for advancing SCLC treatment.

## Introduction

Small cell lung cancer (SCLC) is a highly aggressive malignancy that is associated with exposure to tobacco carcinogens and is characterized by rapid growth, metastasis, and drug resistance (1). SCLC that occurs *de novo* represents about 15% of lung cancers (1). Resistance of non–small cell lung adenocarcinomas (NSCLC) to EGFR inhibitors is also associated with transdifferentiation to SCLCs at rates of 5% to 14% (2). Patients with SCLC have had limited therapeutic options other than standard chemotherapy (1, 3); however, treatment with immune checkpoint inhibitors (ICI) has been associated with response rates of 10% to 14% and increases in survival when combined with chemotherapy (3). Despite these advances, patients diagnosed with SCLC have a median overall survival of ∼1 year, emphasizing the critical need for identifying other effectors that contribute to SCLC progression. SCLCs arise from pulmonary neuroendocrine (NE) stem cells that are activated by lung injury and are regulated by p53, RB, and NOTCH in the wound repair response (4). SCLCs exhibit widespread functional loss of p53 and RB, and dysregulation of the MYC and E2F pathways (1, 5–7). As a result, a notable characteristic of NE SCLC cells is activation of the replication stress response (RSR), which is dependent on DNA repair and cell-cycle checkpoints for proliferation (8–10). NE SCLC subtypes have been characterized by differential expression of the ASCL1 and NEUROD1 transcription factors (TF) and non-NE SCLCs by POU2F3 and YAP1 (1, 11). Otherwise, the identification of potential targets for SCLC treatment has remained a major unmet medical need (6).

MUC1-C appeared in mammals to provide protection of pulmonary and other types of epithelial cells from loss of homeostasis associated with exposure to the external environment (12). MUC1-C induces inflammatory, proliferative, and remodeling signaling pathways that contribute to the wound healing response (12). In addition and in settings of chronic inflammation with repetitive cycles of damage and repair, prolonged MUC1-C activation promotes cancer progression (12). In concert with this capacity for driving oncogenesis, MUC1-C is aberrantly overexpressed in diverse carcinomas and is associated with poor clinical outcomes (12). MUC1-C contributes to multiple hallmarks of the cancer cell, including the epithelial–mesenchymal transition (EMT), cancer stem cell (CSC) state, epigenetic reprogramming, and lineage plasticity (12, 13), which are associated with drug resistance and immune evasion (14). Along these lines, MUC1-C confers resistance of CSCs to genotoxic anticancer agents (12), at least in part, by (i) binding directly to ATM and γH2AX and promoting repair of DNA double-strand breaks (DSB; ref. 15), and (ii) integrating epigenetic reprogramming with activation of PARP in the DNA damage response (DDR; ref. 16). MUC1-C also promotes immune evasion by intrinsic production of effectors that suppress the tumor microenvironment (12, 17).

There is no known involvement of MUC1-C in SCLC. Our studies demonstrate that MUC1-C is of functional importance in SCLC by driving activation of the MYC pathway. We show that MUC1-C→MYC signaling is integrated with induction of E2F target genes and dysregulation of cell-cycle progression. We also show that the MUC1-C→MYC pathway induces (i) NOTCH2, (ii) NE dedifferentiation, and (iii) self-renewal and tumorigenicity. These findings reveal a previously unrecognized addiction to MUC1-C, as defined by dependence for survival (18), in classic, variant and non-NE SCLC subtypes.

## Materials and Methods

### Cell culture

NCI-H69 cells (ATCC; nonsense mutations in TP53 and RB1) and COR-L279 cells (Sigma-Aldrich; deletion mutation in TP53) were cultured in RPMI1640 medium (Corning) supplemented with 10% FBS and 2 mmol/L glutamine. DMS53 cells (ATCC; missense mutation in TP53) were grown in Weymouth medium (Thermo Fisher Scientific) supplemented with 10% FBS. NCI-H526 cells (ATCC; splice acceptor mutation in TP53) were cultured in RPMI1640 medium with 10% FBS. Authentication of the cells was performed by short tandem repeat (STR) analysis. Cells were monitored for mycoplasma contamination using the MycoAlert Mycoplasma Detection Kit (Lonza). Cells were maintained for 3 to 4 months for performing experiments.

### Gene silencing

MUC1shRNA (MISSION shRNA TRCN0000122938; Sigma), MYCshRNA (MISSION shRNA TRCN0000039642; Sigma) or a control scrambled shRNA (CshRNA; Sigma) was inserted into the pLKO-tet-puro vector (Plasmid #21915; Addgene). Single guide RNAs targeting *MUC1* exon 4 were inserted into the lentiCRISPR v2 (Plasmid #52961; Addgene). Single guide RNAs targeting NOTCH2 were inserted into the lentiCRISPR v2 hygro (Plasmid #98291; Addgene). The viral vectors were produced in 293T cells as described in ref. 19. Cells transduced with the vectors were selected for growth in 1 to 4 μg/mL puromycin or 100 to 400 μg/mL hygromycin. Single cell clones were isolated by the array dilution method. Cells were treated with 0.1% DMSO as the vehicle control or 500 ng/mL doxycycline (DOX; Millipore Sigma).

### Quantitative reverse-transcription PCR (qRT-PCR)

Total cellular RNA was isolated for qRT-PCR as described in ref. 19, using primers listed in Supplementary Table S2.

### Immunoblot analysis

Total lysates prepared from subconfluent cells were subjected to immunoblot analysis using anti-ASCL1 (GTX129189; GeneTex), anti-YAP1 [14074; Cell Signaling Technology (CST)], anti-NEUROD1 (4373; CST), anti-POU2F3/SKN1 (ab101724; Abcam), anti-MUC1-C (HM-1630-P1ABX; Thermo Fisher Scientific), anti-GAPDH (5174; CST), anti-β-actin (A5441; Sigma-Aldrich), anti-CDK1 (ab131450; Abcam), anti-CCNA2/cyclin A2 (4656; CST), anti-PLK1 (4513, CST), anti-AURKA (ab1287, Abcam), anti-γH2AX (9718, CST), anti-NOTCH2 (5732; CST), anti-MDK (ab52637, Abcam), anti-BRN2 (12137; CST), and anti-MYC (ab32072; Abcam), anti-HES1 (11988, CST), anti-BMI1 (6964; CST), anti-CD133 (5860; CST) and anti-CD44 (KO601; TransGenic Inc.).

### RNA-seq analysis

Total RNA from cells cultured in triplicates was isolated for generation of the RNA-seq datasets as described in ref. 19. Gene Set Enrichment Analysis (GSEA) was performed as described in ref. 19.

### Tumorsphere formation assays

Cells (1.5–3.0 × 10^4^) were seeded per well in 6-well ultra-low attachment culture plates (Corning Life Sciences) in DMEM/F12 50/50 medium (Corning Life Sciences) with 20 ng/mL EGF (Millipore Sigma), 20 ng/mL bFGF (Millipore Sigma), and 1% B27 supplement (Gibco). In certain studies, cells were treated with (i) vehicle control or 500 ng/mL DOX and (ii) 5 μmol/L GO-203 dissolved in PBS (19–23). Tumorspheres were counted under an inverted microscope in triplicate wells.

### Colony formation assays

Cells were treated with control vehicle or 5 μmol/L GO-203 for 3 days, seeded at 1,500 cells per well in 24-well plates and then stained with 0.5% crystal violet in 25% methanol. Colonies >25 cells were counted in triplicate wells.

### Mouse tumor model studies

Six- to 8-week old nude mice (Taconic Farms) were injected subcutaneously in the flank with 3–5 × 10^6^ cells in 100 μL of a 1:1 solution of medium and Matrigel (BD Biosciences). When the mean tumor volume reached 100 to 150 mm^3^, mice were pair-matched into groups. In studies of H69/tet-MUC1shRNA and COR-L279/tet-MUC1shRNA tumors, mice were fed without or with DOX (625 ppm, every day). Mice bearing established COR-L279 tumors were treated with 5 mg/kg GO-203 administered intraperitoneally each day. Tumor measurements and body weights were recorded twice each week. Mice were sacrificed when tumors reached >2,000 mm^3^, as calculated by the formula: (width)^2^ × length/2. These studies were conducted in accordance with and approved by the Dana-Farber Cancer Institute Animal Care and Use Committee under protocol 03-029.

### IHC

Formalin-fixed, paraffin-embedded (FFPE) sections of SCLC tissue samples were deparaffinized in xylene. Antigen retrieval was performed at 95°C to 99°C for 40 minutes and at room temperature for 20 minutes in pH 8.5 EDTA buffer (E1161, Sigma-Aldrich). Peroxidase blocking (Peroxidazed 1, Biocare Medical) was performed for 5 minutes followed by universal blocking (Background sniper, Biocare Medical) for 10 minutes. Sections were incubated with anti-MUC1-C (dilution 1:100, MA5–11202; Thermo Fisher Scientific) for 2 hours at room temperature, anti-Armenian hamster secondary antibody (dilution 1:200, ab5745; Abcam) for 30 minutes at room temperature and diaminobenzidine tetrahydrochloride chromagen reagents for 5 minutes. Immunostained sections were counterstained with hematoxylin.

### Statistical analysis

Data obtained from experiments performed at least three times are expressed as the mean ± SD. Data were analyzed using the unpaired Mann–Whitney *U* test with *P* values of <0.05 (*) considered statistically significant.

### Analysis of human SCLC tumor datasets

Data analysis was performed using the cBioPortal Cancer Genomic and Oncomine websites (24, 25). GSE60052 was downloaded from Gene Expression Omnibus (GEO; https://www.ncbi.nlm.nih.gov/geo/query/acc.cgi?acc=GSE60052).

GSE138267 is the accession number of the scRNA-seq SCLC dataset (https://www.ncbi.nlm.nih.gov/geo/query/acc.cgi?acc=GSE138474). Processed scRNA-seq data comprising untreated xenograft tissue derived from circulating tumor cells captured from 7 patients with SCLC (25) were obtained from the Gene Expression Omnibus (GSE138474). SCLC cell data were re-analyzed via Seurat (26) for variable feature selection, dimensionality reduction (PCA), and uniform manifold approximation and projection (UMAP) low-dimensional representation using a workflow and parameters described by Stewart and colleagues SCLC cell gene expression was imputed using MAGIC (27), implemented via the Rmagic package. Single-cell pathway enrichment was performed on imputed expression by AUCell (28), using select HALLMARK pathways. Associations between MUC1 expression and select genes or signatures within SCLC cells were examined by Pearson correlation analysis.

### Data availability

The accession numbers for the RNA-seq data are GEO Submissions GSE159801 and GSE192475.

## Results

### MUC1-C regulates the transcriptomes of SCLC-A cell lines

**Figure 1.**
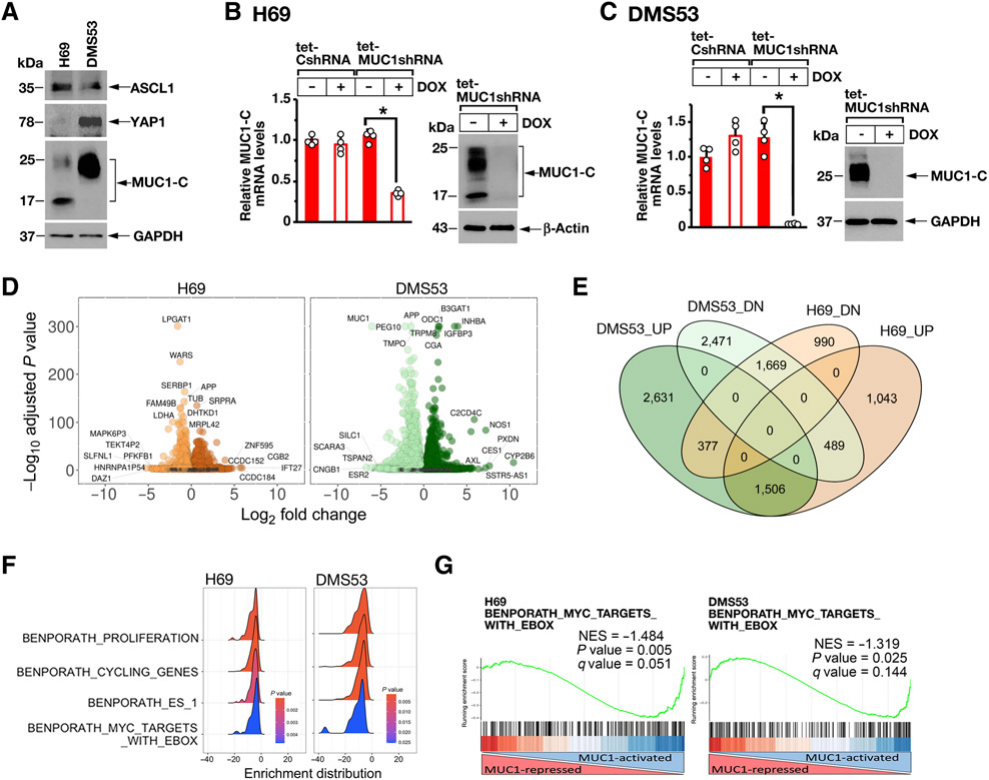
MUC1-C regulates common transcriptomes in SCLC-A cells. **A,** Lysates from H69 and DMS53 cells were immunoblotted with antibodies against the indicated proteins. **B** and **C**. H69 (**B**) and DMS53 (**C**) cells expressing a tet-CshRNA or a tet-MUC1shRNA were treated with vehicle or 500 ng/mL DOX for 7 days. The cells were analyzed for MUC1-C mRNA levels by qRT-PCR using primers listed in Supplementary Table S1. The results (mean ± SD of four determinations) are expressed as relative mRNA levels compared with that obtained for vehicle-treated cells (assigned a value of 1; left). Lysates were immunoblotted with antibodies against the indicated proteins (right). **D,** RNA-seq was performed in triplicate on H69/tet-MUC1shRNA and DMS53/tet-MUC1shRNA cells treated with vehicle or DOX for 7 days. The datasets were analyzed for effects of MUC1-C silencing on repressed and activated genes as depicted by the Volcano plots. **E,** Overlap of down- and upregulated genes in H69 and DMS53 cells with MUC1-C silencing. **F,** RNA-seq datasets from H69 (left) and DMS53 (right) cells were analyzed with GSEA for enrichment distribution using the indicated BENPORATH collection gene signatures. **G,** RNA-seq datasets from H69 (left) and DMS53 (right) cells were analyzed with GSEA using the BENPORATH MYC TARGETS WITH EBOX gene signatures.

The human NCI-H69 SCLC cell line is characteristic of the SCLC-A classic subtype that expresses ASCL1 and predominantly grows as aggregates of floating cells (Fig. 1A; ref. 1). Human DMS53 SCLC cells grow largely as adherent monolayers and express ASCL1, consistent with the NE SCLC-A subtype (Fig. 1A; ref. 1). DMS53 cells also express YAP1, which has been questioned as another marker of SCLC subtypes (1). In H69 cells, MUC1-C is expressed as N-glycosylated 25 to 20 kDa and unglycosylated ∼17 kDa MUC1-C proteins (Fig. 1A). Expression of MUC1-C in DMS53 cells is predominantly the 25 to 20 kDa form (Fig. 1A). To assess potential MUC1-C functions, we established H69 cells stably expressing a tet-inducible control shRNA (tet-CshRNA) or a tet-MUC1shRNA. Treatment with doxycycline (DOX) was associated with downregulation of MUC1-C in H69/tet-MUC1shRNA, and not H69/tet-CshRNA, cells (Fig. 1B, left and right). Similar results were obtained from DOX-treated DMS53/tet-MUC1shRNA cells (Fig. 1C, left and right), supporting potential models for RNA-seq studies to assess MUC1-C-driven changes in gene expression. Along these lines, volcano plots of the RNA-seq datasets from H69 and DMS53 cells with MUC1-C silencing revealed substantial effects on repressed and activated genes (Fig. 1D, left and right), among which we identified 1,634 downregulated and 1,410 upregulated genes that are common to both cell lines (Fig. 1E). Analysis by GSEA using the BENPORATH collection identified MUC1-C–regulated signatures associated with proliferation, cell-cycle progression and the embryonic stem cell (ESC) state (Fig. 1F). We also found that silencing MUC1-C in H69 and DMS53 cells associates with downregulation of the BENPORATH MYC TARGETS WITH EBOX gene signature (Fig. 1F and G, left and right; Supplementary Fig. S1A) and MYC target genes that are expressed in both cell lines (Supplementary Fig. S1B).

### MUC1-C regulates common MYC target genes in classic, variant, and non-NE SCLC cells

**Figure 2.**
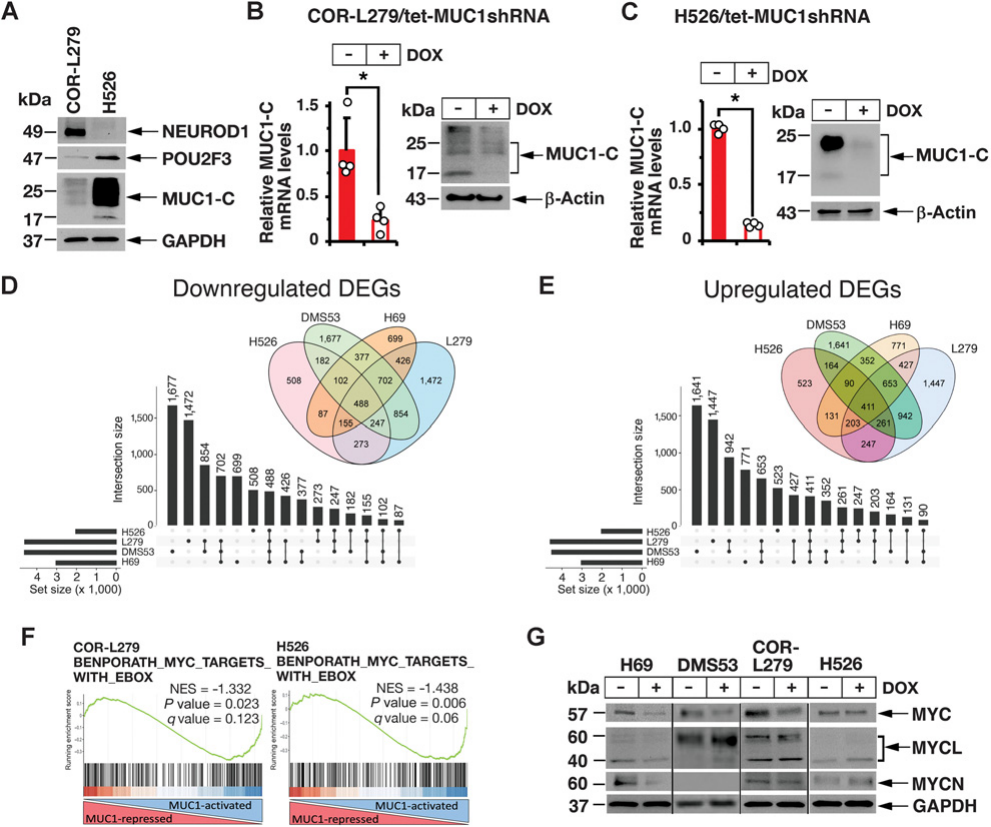
MUC1-C regulates common and distinct transcriptomes in NE and non-NE SCLC subtypes. **A,** Lysates from COR-L279 and H526 cells were immunoblotted with antibodies against the indicated proteins. **B** and **C**. COR-L279 (**B**) and H526 (**C**) cells expressing a tet-CshRNA or a tet-MUC1shRNA were treated with vehicle or 500 ng/mL DOX for 7 days. The cells were analyzed for MUC1-C mRNA levels by qRT-PCR. The results (mean ± SD of four determinations) are expressed as relative mRNA levels compared with that obtained for vehicle-treated cells (assigned a value of 1; left). Lysates were immunoblotted with antibodies against the indicated proteins (right). **D** and **E.** RNA-seq analysis was performed in triplicate on COR-L279/tet-MUC1shRNA and H526/tet-MUC1shRNA cells treated with vehicle or DOX for 7 days. Common DEGs downregulated (**D**) and upregulated (**E**) by MUC1-C silencing in the indicated SCLC cells. The lollipop plots show common numbers of DEGs in the four different SCLC cells. **F,** RNA-seq datasets from the indicated cells were analyzed with GSEA using the BENPORATH MYC TARGETS WITH EBOX. **G,** The designated SCLC cells expressing a tet-MUC1shRNA were treated with vehicle of DOX for 7 days. Lysates were immunoblotted with antibodies against the indicated proteins.

To extend these results to other SCLC subtypes, we studied (i) variant SCLC-N COR-L279 cells that express NEUROD1, and exhibit characteristics of NE differentiation, and (ii) SCLC-P H526 cells that express POU2F3, but not the NE phenotype (Fig. 2A; ref. 1). MUC1-C was detectable in COR-L279 cells and at higher levels in H526 cells (Fig. 2A). Accordingly, we silenced MUC1-C in these cells (Fig. 2B and C) and by RNA-seq analysis (Supplementary Fig. S2A) identified 488 downregulated and 411 upregulated differentially expressed genes (DEG) common to H69, DMS53, COR-L279, and H526 cells (Fig. 2D and E). Consistent with findings in H69 and DMS53 cells, RNA-seq analysis of COR-L279 and H526 cells further demonstrated that MUC1-C is necessary for activation of the BENPORATH MYC TARGETS WITH EBOX signature (Fig. 2F, left and right). Shared sets of MUC1-C–regulated MYC target genes were identified COR-L279 and H526 cells (Supplementary Fig. S2B). Moreover, MUC1-C was necessary for the upregulation and downregulation of common sets of MYC target genes in H69, DMS53, COR-L279, and H526 cells (Supplementary Figs. S2C and S2D). MUC1-C activates MYC expression by a WNT pathway-mediated mechanism that is dependent on cancer cell context (12). MUC1-C also binds directly to the MYC HLH-LZ domain to drive MYC target genes (29). MYC, MYCL, and MYCN are expressed in the major SCLC subtypes (1). We found that silencing MUC1-C results in the downregulation of MYC in H69, DMS53, and COR-L279 cells (Fig. 2G). MUC1-C was also necessary for expression of MYCL and MYCN in H69 cells, but not in DMS53, COR-L279, and H526 cells (Fig. 2G). Accordingly, we focused our studies on MUC1-C–induced regulation of the MYC pathway.

### MUC1-C drives cell-cycle progression and DNA RSR in SCLC cells

MYC cooperates with loss of RB in driving SCLC proliferation (5). Analysis of the H69, DMS53, and COR-L279 cell RNA-seq datasets revealed that MUC1-C significantly associates with downregulation of the BENPORATH CYCLING GENES (Supplementary Fig. S3A) and GO CELL DIVISION gene signatures (Supplementary Fig. S3B). In contrast, analysis of the RNA-seq datasets from non-NE H526 cells revealed upregulation of these cell-cycle–related gene signatures (Supplementary Figs. S3A and S3B). To extend the potential involvement of MUC1-C in driving SCLC cell-cycle progression, we focused on H69 and DMS53 cells and found that MUC1-C induces expression of CCNA2 (cyclin A2) and CDK1, which in concert regulate entry into mitosis (Supplementary Fig. S3C; ref. 30). To confirm these results, we targeted MUC1-C with CRISPR/Cas9 editing using two different MUC1sgRNAs and found that MUC1-C drives CCNA2 and CDK1 expression (Supplementary Fig. S3D). Analysis of MUC1-C-driven genes also uncovered *PLK1* and *AURKA* (Supplementary Fig. S3E), which are of significance in that their encoded proteins have been identified as potential targets for SCLC treatment (9). PLK1 plays a role in the DNA RSR and regulation of the G_2_–M checkpoint (30). Aurora kinase A activates PLK1 and protects DNA forks during replicative stress (30). In addition, PLK1 and aurora kinase A function with CDK1 in regulating mitotic progression (30). Consistent with these functions, silencing MUC1-C with downregulation of the cyclin A2, CDK1, PLK1, and aurora kinase A proteins was associated with induction of DNA replication stress as evidenced by increases in γH2AX (Supplementary Fig. S3F, left and right) and delays in G_2_–M phase progression (Supplementary Fig. S3G). GSEA analysis further demonstrated that MUC1-C significantly associates with activation of the RSR DEFECT GENE SIGNATURE (Supplementary Fig. S3H) and suppression of the GO REGULATION OF RESPONSE TO DNA DAMAGE STIMULUS gene signature (Supplementary Fig. S3I). These findings collectively indicated that MUC1-C regulates (i) MYC signaling in both NE and non-NE SCLC subtypes, and (ii) cell-cycle pathways in SCLC cells that have been associated with NE differentiation and replication stress (10).

### MUC1-C→MYC signaling is necessary for NOTCH2 expression

**Figure 3.**
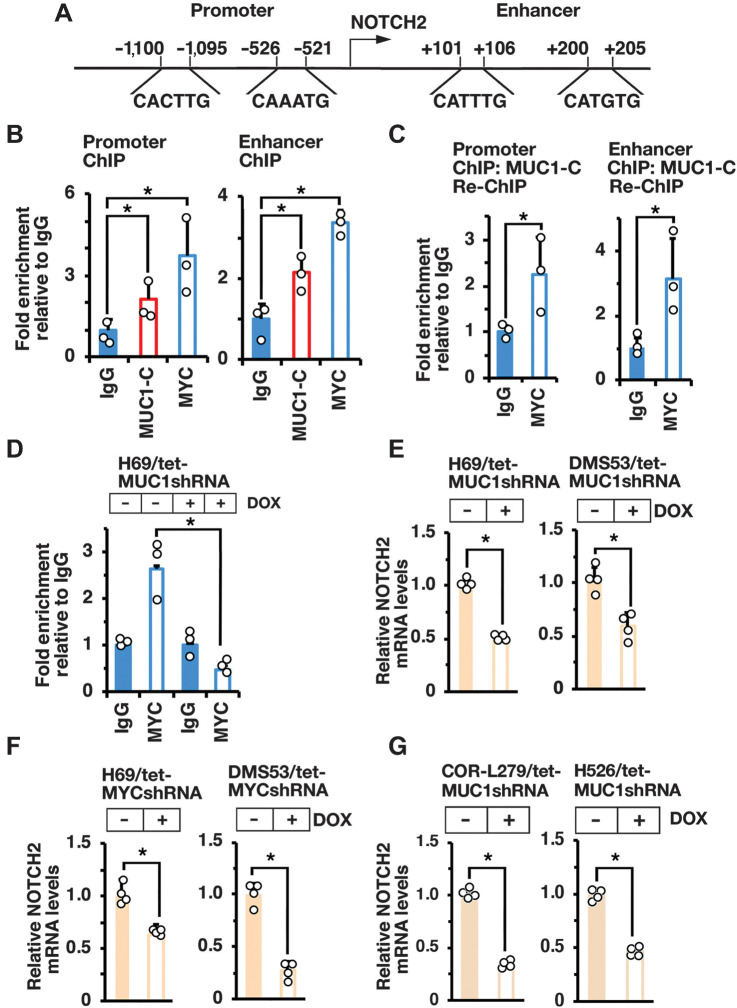
MUC1-C activates NOTCH2 expression by a MYC-mediated mechanism. **A,** Schema of the *NOTCH2* promoter and enhancer regions with locations of potential E-boxes. **B** and **C**. Soluble chromatin from H69 cells was precipitated with anti-MUC1-C, anti-MYC, or a control IgG (**B**). Soluble chromatin was precipitated with anti-MUC1-C (ChIP) and then reprecipitated with anti-MYC or a control IgG (re-ChIP; **C**). The DNA samples were amplified by qPCR with primers for the *NOTCH2* promoter and enhancer regions. **D,** Soluble chromatin from H69/tet-MUC1shRNA cells treated with vehicle or DOX for 7 days was precipitated with anti-MYC or a control IgG. The DNA samples were amplified by qPCR with primers for the *NOTCH2* promoter region. The results (mean ± SD of three determinations) are expressed as the relative fold enrichment compared with that obtained with the IgG control (assigned a value of 1). **E,** H69/tet-MUC1shRNA (left) and DMS53/tet-MUC1shRNA (right) cells treated with vehicle or DOX for 7 days were analyzed for NOTCH2 mRNA levels by qRT-PCR. **F,** H69/tet-MYCshRNA (left) and DMS53/tet-MYCshRNA (right) cells treated with vehicle or DOX for 7 days were analyzed for NOTCH2 mRNA levels by qRT-PCR. **G,** COR-L279/tet-MUC1shRNA (left) and H526/tet-MUC1shRNA (right) cells treated with vehicle or DOX for 7 days were analyzed for NOTCH2 mRNA levels by qRT-PCR. The results (mean ± SD of four determinations) are expressed as relative mRNA levels compared with that obtained for vehicle-treated cells (assigned a value of 1).

GSEA of the RNA-seq datasets using the BENPORATH_ES1 signature, which is derived from genes enriched in ESCs and CSC-like phenotypes (Ben-Porath I, Nat. Genet. 2008], revealed that MUC1-C associates with the regulation of NOTCH signaling. Along these lines, we found that MUC1-C drives expression of (i) NOTCH2 in H69, DMS53, COR-L279, and H526 cells; (ii) NOTCH1 in DMS53 and H526 cells; and (iii) NOTCH3 in DMS53 and COR-L279 cells (Supplementary Figs. S4A–S4D). NOTCH2 is a marker of NE stem cells, which initiate NE reprogramming after injury and are the proposed origin of SCLC (4). On the basis of MUC1-C–induced regulation of MYC target genes in H69, DMS53, COR-L279, and H526 cells, we analyzed *NOTCH2* for potential MYC binding motifs and identified E-boxes in the promoter and proximal enhancer regions (Fig. 3A). Consistent with MUC1-C binding directly to the MYC HLH-LZ domain (29), we performed ChIP studies that demonstrated occupancy of MUC1-C and MYC on the *NOTCH2* promoter region (Fig. 3B, left). Similar results were obtained from ChIP analysis of the *NOTCH2* proximal enhancer region (Fig. 3B, right). Re-ChIP analysis further demonstrated that MUC1-C forms a complex with MYC on both regions (Fig. 3C, left and right). Silencing MUC1-C was associated with decreases in MYC occupancy (Fig. 3D) and downregulation of NOTCH2 expression (Fig. 3E, left and right). Moreover, silencing MYC decreased NOTCH2 mRNA levels (Fig. 3F, left and right). In concert with the demonstration that MUC1-C is necessary for activation of MYC target genes in COR-L279 and H526 cells, silencing MUC1-C in these models also resulted in suppression of NOTCH2 transcripts (Fig. 3G, left and right), in support of a MUC1-C→MYC→NOTCH2 pathway pathway in NE and non-NE SCLC cells.

### MUC1-C→MYC signaling integrates induction of NOTCH2 and NE dedifferentiation

**Figure 4.**
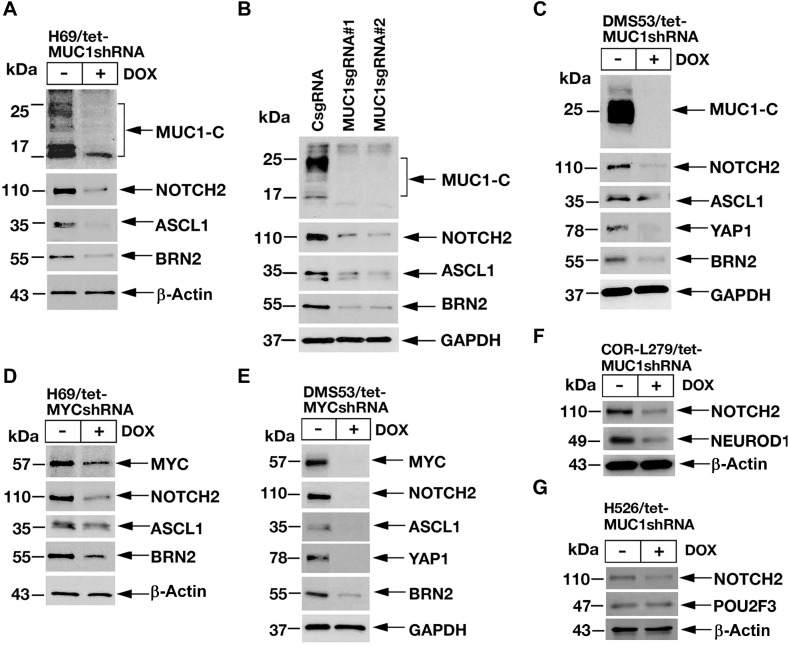
Targeting MUC1-C suppresses ASCL1, NEUROD1, and YAP1 expression. **A,** Lysates from H69/tet-MUC1shRNA cells treated with vehicle or DOX for 7 days were immunoblotted with antibodies against the indicated proteins. **B,** Lysates from H69/CsgRNA, H69/MUC1sgRNA#1, and H69/MUC1sgRNA#2 cells were immunoblotted with antibodies against the indicated proteins. **C,** Lysates from DMS53/tet-MUC1shRNA cells treated with vehicle or DOX for 7 days were immunoblotted with antibodies against the indicated proteins. **D** and **E,** Lysates from H69/tet-MYCshRNA (**D**) and DMS53/tet-MYCshRNA (**E**) cells treated with vehicle or DOX for 7 days were immunoblotted with antibodies against the indicated proteins. **F** and **G,** Lysates from COR-L279/tet-MUC1shRNA (**F**) and H526/tet-MUC1shRNA (**G**) cells treated with vehicle or DOX for 7 days were immunoblotted with antibodies against the indicated proteins.

SCLC subtypes have been defined by differential expression of the ASCL1, NEUROD1, POU2F3, and YAP1 TFs (1, 11). In classic SCLC-A cells, ASCL1 is a key determinant of NE cell differentiation (1). In addition to NOTCH2, silencing MUC1-C in H69 cells resulted in suppression of ASCL1, as well as POU3F2/BRN2, another neural TF that promotes NE differentiation (Fig. 4A; ref. 31). These findings were extended with similar results in H69/MUC1sgRNA cells; that is targeting MUC1-C decreases NOTCH2, ASCL1, and BRN2 (Fig. 4B). DMS53 cells express ASCL1, as well as the YAP1 TF, which is activated by the HIPPO pathway (1). In DMS53 cells, we found that silencing MUC1-C is associated with decreases in NOTCH2, ASCL1, YAP1, and BRN2 (Fig. 4C). The importance of the MUC1-C→MYC pathway was further supported by the demonstration that silencing MYC in H69 (Fig. 4D) and DMS53 (Fig. 4E) cells also results in suppression of NOTCH2, ASCL1, YAP1, and BRN2. In extending this analysis to SCLC-N COR-L279 cells, we found that MUC1-C is necessary for expression of NOTCH2 and NEUROD1 (Fig. 4F). In H526 cells, silencing MUC1-C decreased NOTCH2 levels, but had little if any effect on POU2F3 expression (Fig. 4G). These findings supported involvement of MUC1-C→MYC signaling in integrating activation of the NOTCH2 pathway and dedifferentiation with induction of the ASCL1 and NEUROD1 TFs.

### MUC1-C→MYC→NOTCH2 signaling drives SCLC cell self-renewal capacity

**Figure 5.**
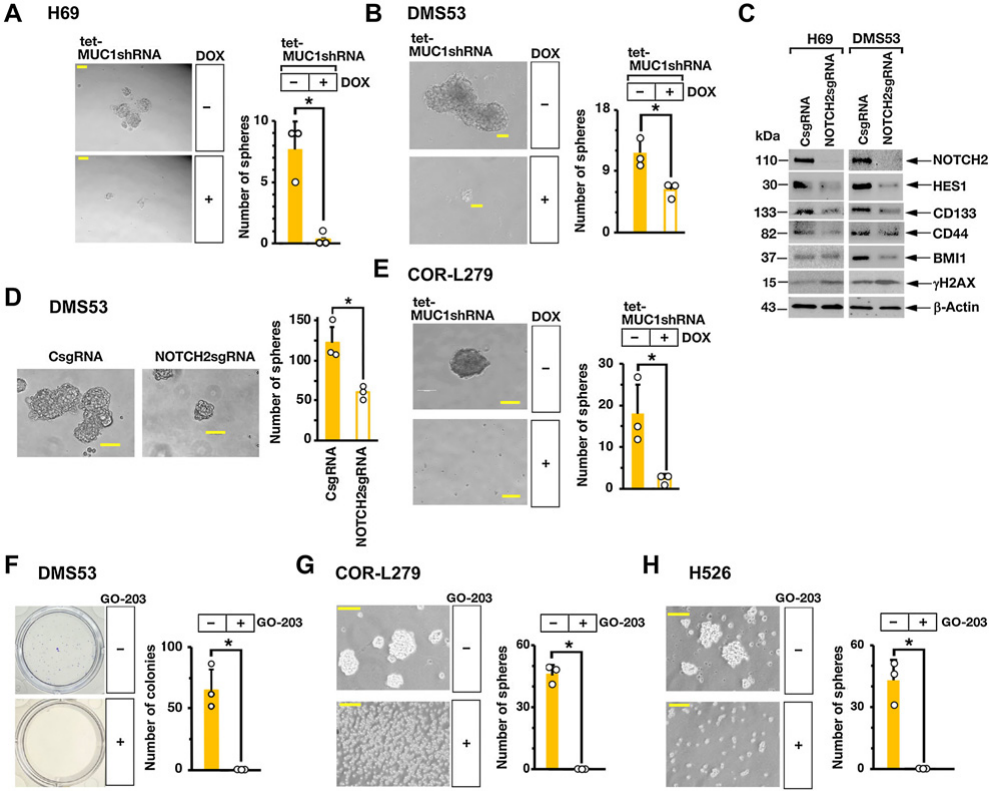
MUC1-C is necessary for SCLC cell self-renewal. **A** and **B,** Representative images of tumorspheres derived from H69/tet-MUC1shRNA (**A**) and DMS53/tet-MUC1shRNA (**B**) cells treated with control vehicle or DOX for 7 days (left). Bar represents 50 μm. The number of tumorspheres is expressed as the mean ± SD of three determinations (right). **C,** Lysates from H69 (left) and DMS53 (right) cells expressing a CsgRNA or NOTCH2sgRNA were immunoblotted with antibodies against the indicated proteins. **D,** Representative images of tumorspheres derived from DMS53/CshRNA and DMS53/NOTCH2shRNA cells (left). Bar represents 50 μm. The number of tumorspheres is expressed as the mean ± SD of three determinations (right). **E,** Representative images of tumorspheres derived from COR-L279/tet-MUC1shRNA cells treated with control vehicle or DOX for 7 days (left). Bar represents 50 μm. The number of tumorspheres is expressed as the mean ± SD of three determinations (right). **F,** DMS53 cells treated with control vehicle or 5 μmol/L GO-203 for 3 days were seeded in dishes for 12 days. Representative images are shown for colonies stained with crystal violet (left). The number of colonies is expressed as the mean ± SD of three determinations (right). **G** and **H,** Representative images of tumorspheres derived from COR-L279 (**G**) and H526 (**H**) cells treated with control vehicle or 5 μmol/L GO-203 for 7 days (left). Bar represents 50 μm. The number of tumorspheres is expressed as the mean ± SD of three determinations (right).

In extending involvement of the MUC1-C→MYC→NOTCH2 pathway in SCLC dedifferentiation, we found that silencing MUC1-C in H69 (Fig. 5A, left and right) and DMS53 (Fig. 5B, left and right) cells suppresses self-renewal capacity as assessed by decreases in tumorsphere formation. We also found that silencing NOTCH2 in H69 and DMS53 cells is associated with downregulation of (i) the NOTCH target gene, HES1, which like NOTCH2 contributes to stemness (32), and (ii) the CD133, CD44, and BMI1 stemness factors (Fig. 5C, left and right). In addition, silencing NOTCH2 resulted in induction of γH2AX expression (Fig. 5C, left and right), in concert with intersection of the RSR and CSC state (33, 34) and suppressed self-renewal capacity (Fig. 5D, left and right). In extending this work to variant COR-L279 cells, we found that silencing MUC1-C similarly inhibits self-renewal capacity (Fig. 5E). As an additional approach, we treated SCLC cells with the GO-203 inhibitor, which is a cell-penetrating peptide that blocks MUC1-C homodimerization, nuclear localization, and function (12, 20–23). Treatment of H69, DMS53, COR-L279, and H526 cells with GO-203 demonstrated similar effects on (i) viability with IC_50_ values of ∼2 to 4 μmol/L and (ii) proliferation and (iii) induction of cell death (Supplementary Figs. S5A–S5H). Moreover and of importance, GO-203 was effective in suppressing SCLC cell capacity for colony formation and self-renewal (Fig. 5F–H, left and right).

### MUC1-C→MYC→NOTCH2 pathway is necessary for SCLC tumorigenicity

**Figure 6.**
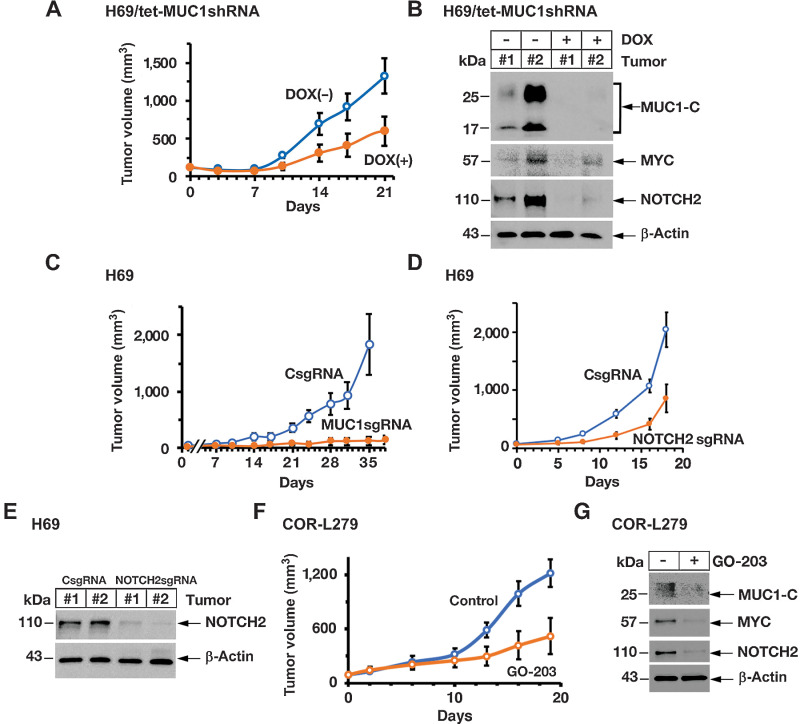
Targeting MUC1-C suppresses SCLC tumorigenicity. **A,** Six-week old nude mice were injected subcutaneously in the flank with 1 × 10^6^ H69/tet-MUC1shRNA cells. Mice were pair-matched into two groups when tumors reached 100 to 150 mm^3^ and were fed without and with DOX. Tumor volumes are expressed as the mean ± SD for 6 mice. **B,** Lysates from two untreated and two DOX-treated H69/tet-MUC1shRNA tumors obtained on day 21 were immunoblotted with antibodies against the indicated proteins. **C,** Six-week-old nude mice were injected subcutaneously in the flank with 1 × 10^6^ H69/CsgRNA and H69/MUC1sgRNA cells. Tumor volumes are expressed as the mean ± SD for 6 mice. **D,** Six-week-old nude mice were injected subcutaneously in the flank with 1 × 106 H69/CsgRNA and H69/NOTCH2sgRNA cells. Tumor volumes are expressed as the mean ± SD for 6 mice. **E,** Lysates from two H69/CsgRNA and two H69/NOTCH2sgRNA tumors obtained on day 18 were immunoblotted with antibodies against the indicated proteins. **F** and **G,** Six-week-old nude mice were injected subcutaneously in the flank with 3 × 10^6^ COR-L279 cells. Mice pair-matched into two groups when tumors reached 100 to 150 mm^3^ were treated intraperitoneally each day with PBS or GO-203 for 19 days. Tumor volumes are expressed as the mean ± SEM for 6 mice (**F**). Lysates from tumors harvested on day 19 were immunoblotted with antibodies against the indicated proteins (**G**).

To determine if the MUC1-C→MYC→NOTCH2 pathway is also of importance in promoting tumorigenicity, we fed mice bearing established H69/tet-MUC1shRNA tumors with DOX to silence MUC1-C expression. DOX treatment was associated with significant inhibition of tumor growth (Fig. 6A). Analysis of tumors from control and DOX-treated mice demonstrated downregulation of MUC1-C, as well as MYC and NOTCH2, expression (Fig. 6B). In addition, targeting MUC1-C with a MUC1sgRNA, which suppressed MYC and NOTCH2 *in vitro*, resulted in complete inhibition of tumor growth (Fig. 6C). By extension, silencing NOTCH2 also decreased H69 tumor growth (Fig. 6D and E). As a second model, we confirmed that DOX treatment of mice with COR-L279/tet-MUC1shRNA tumors results in suppression of MUC1-C signaling and tumor growth (Supplementary Figs. S6A and S6B). Moreover, for potential translational relevance, we treated mice bearing established COR-L279 tumors with GO-203 and found inhibition of tumor growth in association with downregulation of MUC1-C, MYC, NOTCH2, and NEUROD1 (Fig. 6F and G), indicating that the MUC1-C→MYC→NOTCH2 pathway is necessary for conferring SCLC tumorigenicity.

### Expression of MUC1 in individual SCLC tumor cells

**Figure 7.**
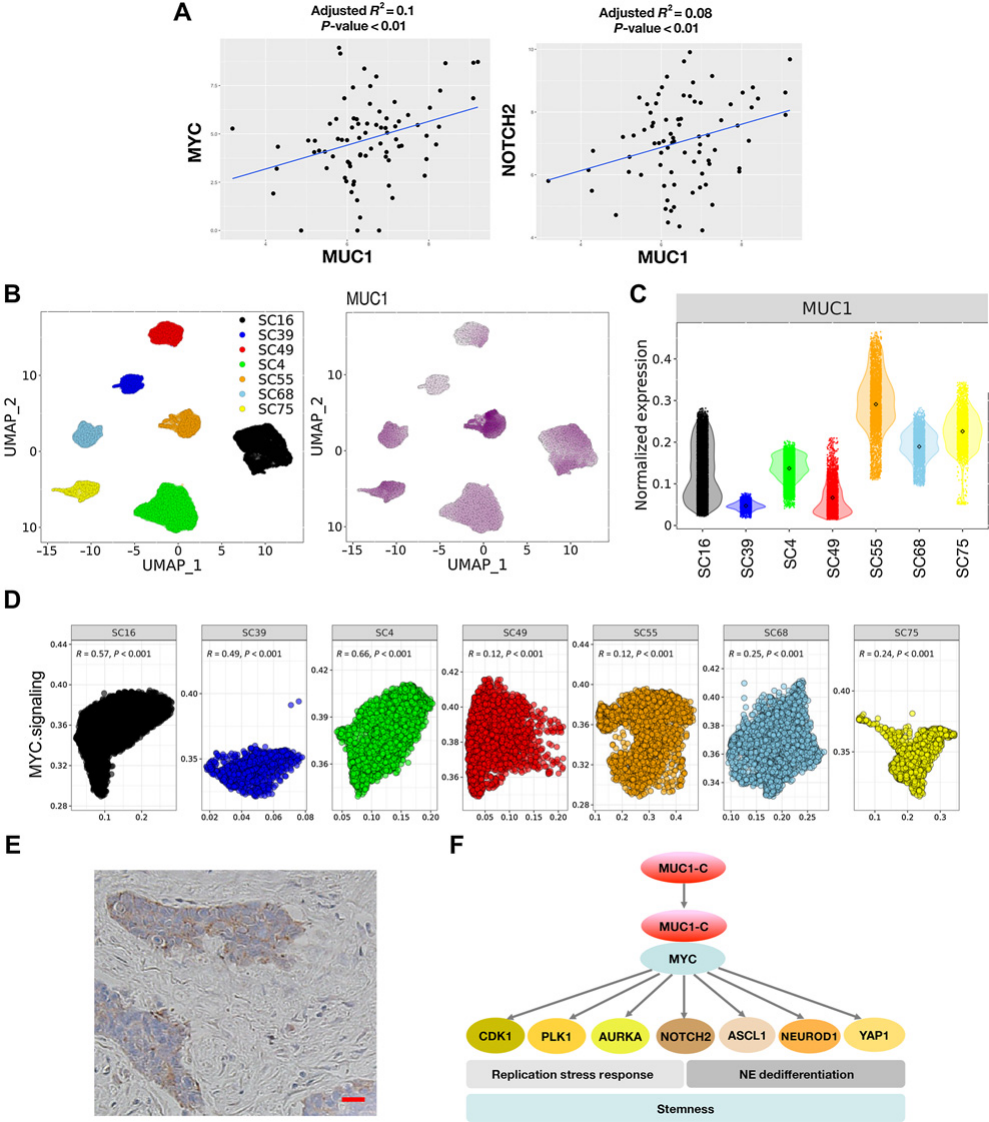
Expression of MUC1 in SCLC tumor tissues. **A,** Analysis of the GSE60052 SCLC bulk RNA-seq dataset demonstrating significant correlations of MUC1 with MYC and NOTCH2 expression in SCLC tumor tissues. **B,** Analysis of the GSE138267 SCLC scRNA-seq dataset demonstrating MUC1 expression in clusters of SCLC cells obtained from seven patient-derived CDX models. **C,** Distribution of MUC1 expression in the seven CDX models. **D,** Significant associations of MUC1 expression with activation of the HALLMARK MYC TARGETS V1 pathway in individual tumors. **E,** Representative IHC staining of MUC1-C expression in SCLC tumor tissue. Bar represents 50 μm. **F,** Proposed model for MUC1-C in driving SCLC progression. MUC1-C activates MYC and E2F target genes that contribute to the RSR and mitotic progression. MUC1-C→MYC signaling also drives expression of NOTCH2 and NE dedifferentiation. MUC1-C thereby integrates activation of the dysregulated MYC and E2F pathways and RSR with NE dedifferentiation, stemness, and self-renewal in SCLC cells.

In extending these findings to SCLC tumor samples, we first analyzed the GSE60052 bulk RNA-seq dataset derived from 79 previously untreated patients (24). In support of our *in vitro* studies, we found that expression of MUC1 in these tumors significantly correlates with MYC and NOTCH2 (Fig. 7A). Further analysis was performed using the scRNA-seq dataset generated from SCLC circulating tumor cells obtained from 7 patients and grown as xenografts (CDX; ref. 25). The datasets were filtered, normalized, and clustered by UMAP (Fig. 7B, left). MUC1 expression was detectable in each of the seven tumor clusters (Fig. 7B, right). By extension, we found that normalized MUC1 expression was uniform within tumors with SC55 exhibiting higher and SC39 lower MUC1 levels (Fig. 7C). In concordance with our *in vitro* findings, MUC1 expression in single SCLC tumor cells was significantly associated with activation of the HALLMARK MYC TARGETS V1 gene signature (Fig. 7D). Consistent with these results, MUC1-C expression was detectable in individual SCLC cells by IHC staining of tumor tissue (Fig. 7E).

## Discussion

The *MUC1* gene evolved in mammals to protect epithelial cells, including those lining the respiratory tract, against loss of homeostasis resulting from exposure to the external environment (12). The MUC1-C subunit contributes to the repair of epithelial cell damage by activating signals associated with inflammatory, proliferative and reprogramming phases of the wound healing response, which if prolonged can promote oncogenesis (12). MUC1 is upregulated in pulmonary epithelial cells exposed to tobacco carcinogens (35). Smoking is a major risk factor for the development of SCLC (1, 3); however, there has been no known involvement of MUC1-C in SCLC progression. Our studies demonstrate that expression of MUC1-C in classic NE SCLC-A, variant NE SCLC-N, and non-NE SCLC-P cells activates the MYC pathway, which is dysregulated in SCLC cells and promotes tumor progression, drug resistance, and poor clinical outcomes (1). In NE SCLC cells, we also found that MUC1-C drives the E2F pathway, which is aberrantly activated by mutational loss of the *RB1* tumor suppressor gene and thereby derepression of E2F-driven gene transcription (1). These findings uncover a previously unrecognized role for MUC1-C in integrating dysregulation of the (i) MYC pathway in NE and non-NE SCLC cells and (ii) E2F pathway in NE SCLC cells (Fig. 7F). MUC1-C has also been linked to integrating the induction of MYC and E2F1 target genes that encode the EZH2/PRC2, BMI1/PRC1, BAF, and PBAF complexes and contribute to MUC1-C–induced epigenetic reprogramming in the DDR and CSC state (13, 16, 36, 37).

Functional loss of p53 and RB with dysregulation of the MYC and E2F pathways and unrestrained proliferation of NE SCLC cells promotes stalled replication forks and activation of the RSR (8–10). NE SCLC cells are dependent on the RSR to protect against genome instability before completion of mitosis (8–10). MYC functionally promotes E2F activity by inducing the expression of cyclins and CDKs that contribute to DNA damage and cell-cycle checkpoints (38–40). We identified *CCNA2* and *CDK1* as MUC1-C-driven MYC and E2F target genes in SCLC cells (Fig. 7F). Cyclin A2 activates CDK1, which promotes progression through G_2_–M phase (30). We also found that MUC1-C induces (i) PLK1, which is dysregulated in SCLC and is a target for SCLC treatment (41) and (ii) aurora kinase A, which has been targeted in MYC amplified SCLC cells with alisertib and other aurora kinase inhibitors (Fig. 7F; ref. 5). PLK1 is activated in the G_2_–M DNA damage repair pathway and in conjunction with CDK1 is necessary for triggering mitosis (30). Aurora kinase A also contributes to the G_2_–M transition by promoting centrosome maturation and mitotic spindle assembly (30). Taken together, these results support involvement of MUC1-C in activating MYC and E2F target genes that promote dysregulation of mitotic progression. In concert with these findings and evidence that SCLC NE differentiation associates with increases in replication stress (10), we found that silencing MUC1-C in NE SCLC cells (i) promotes DSB formation as evidenced by increases in γH2AX, (ii) delays G_2_–M phase progression, and (iii) downregulates G_2_–M and mitotic spindle checkpoint gene signatures.

Epithelial wound healing includes phases of inflammation, proliferation, and remodeling (12, 42). In this regard, pulmonary NE stem cells, which proliferate in the response to injury to promote repair, have been linked to the development of SCLC by constitutive activation of self-renewal (4). In addition, damage to the pulmonary epithelium is associated with reprogramming of NE stem cells and the induction of NOTCH2 as a marker of stemness in the repair response (4). We found that MUC1-C→MYC signaling induces NOTCH2 in NE and non-NE SCLC subtypes (Fig. 7F). MUC1-C/MYC complexes were detectable on the *NOTCH2* promoter and proximal enhancer regions and MUC1-C was necessary for MYC occupancy, consistent with MUC1-C functioning in the activation of MYC target genes (29). We also found that MUC1-C→MYC signaling is associated with induction of ASCL1 and NEUROD1, as well as YAP1, which regulates expression of NE-associated genes (Fig. 7F; refs. 1, 11). These findings support an essential role for MUC1-C in MYC driven SCLC NE dedifferentiation (Fig. 7F; ref. 43). The activities of MYC family members have been ascribed to the regulation of (i) specific subsets of genes, and (ii) the majority of gene promoters in licensing elongation of all RNA PolII complexes, which can pose challenges in the analysis of RNA-seq data (44, 45). Along these lines, cell number-normalized RNA-seq, Ser2PolII immunoblots, and nascent transcription techniques can be employed to address these challenges. In this work, we confirmed our RNA-seq data by demonstrating that MUC1-C and MYC are indeed necessary for activation of the *NOTCH2* stemness-associated gene. We found that targeting MUC1-C genetically as well as pharmacologically with the GO-203 inhibitor suppresses NE and non-NE SCLC cell self-renewal and tumorigenicity. Targeting MYC and NOTCH2 also decreased the capacity for self-renewal and tumor formation corroborating the importance of the MUC1-C→MYC→NOTCH2 pathway in driving the SCLC CSC state. Of note, MUC1 is not designated in DepMap as a common essential gene in SCLC cells. Our results show that targeting MUC1 genetically slows G_2_–M cell-cycle progression, which apparently is insufficient to significantly score in DepMap. Nonetheless and importantly, targeting MUC1 with different MUC1shRNAs and MUC1sgRNAs inhibits self-renewal capacity as determined by tumorsphere formation and tumorigenicity that are not scored by DepMap.

Our findings that MUC1 significantly associates with MYC and NOTCH2 in SCLC tumors further indicate that MUC1-C may be a potential target for inhibiting CSC self-renewal in the treatment of NE and non-NE SCLCs. Of translational relevance, antibodies generated against the MUC1-C extracellular domain have been advanced for clinical evaluation as CAR-T cells and are being developed preclinically as antibody–drug conjugates (46). In dose escalation clinical trials, the MUC1-C GO-203 inhibitor was well-tolerated at low μmol/L plasma levels and, because of a short circulating half-life, is being reformulated in nanoparticles for sustained administration (47). Additional studies will now be needed that determine the effectiveness of targeting MUC1-C in settings of SCLC cells that are resistant to treatment with cytotoxic agents, such as cisplatin and etoposide. Nonetheless, the present findings and the availability of anti-MUC1-C agents could provide new therapeutic options for patients with SCLC.

## Supplementary Material

Supplementary Figure
