# Supplementary material for: Dependence on the MUC1-C Oncoprotein in Classic, Variant, and Non–neuroendocrine Small Cell Lung Cancer
Source: Mol Cancer Res. 2022 May 25;20(9):1379–90. doi: 10.1158/1541-7786.MCR-22-0165 (PMC9437561; doi:10.1158/1541-7786.MCR-22-0165)
Supplement: Supplementary Figure [file mcr-22-0165_supplementary_figure_s1-s6_and_table_s1-s3.suppsm1.pdf]

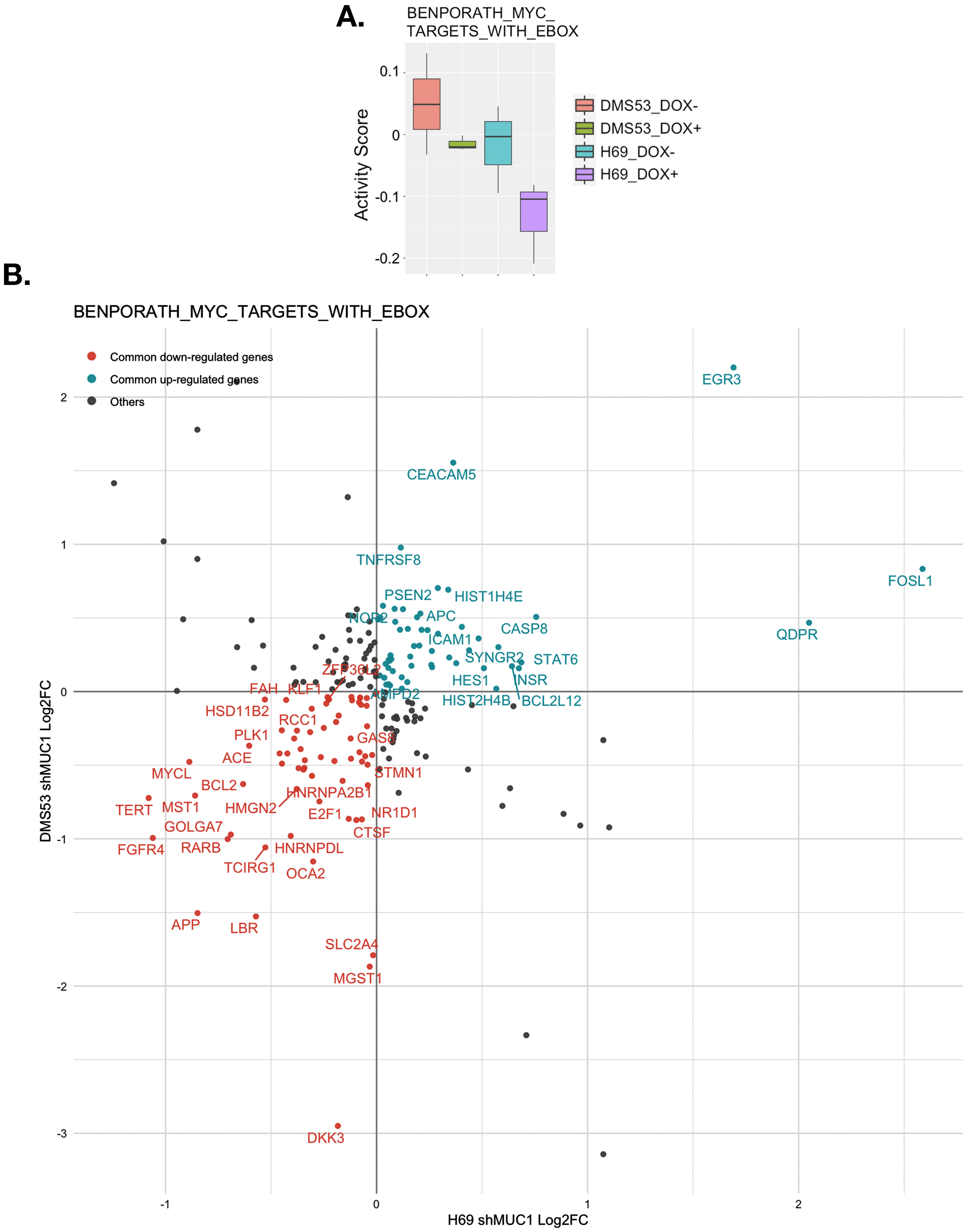

Supplemental Figure S1. MUC1-C regulates MYC target genes in SCLC-A H69 and DMS53 cells. A. GSVA depicting the effects of MUC1

silencing on the BENPORATH MYC TARGETS WITH EBOX gene signature activity scores in H69 and DMS53 cells. **B.** Overlap of the MUC1-C-regulated BENPORATH MYC TARGETS WITH EBOX genes in H69 and DMS53 cells.

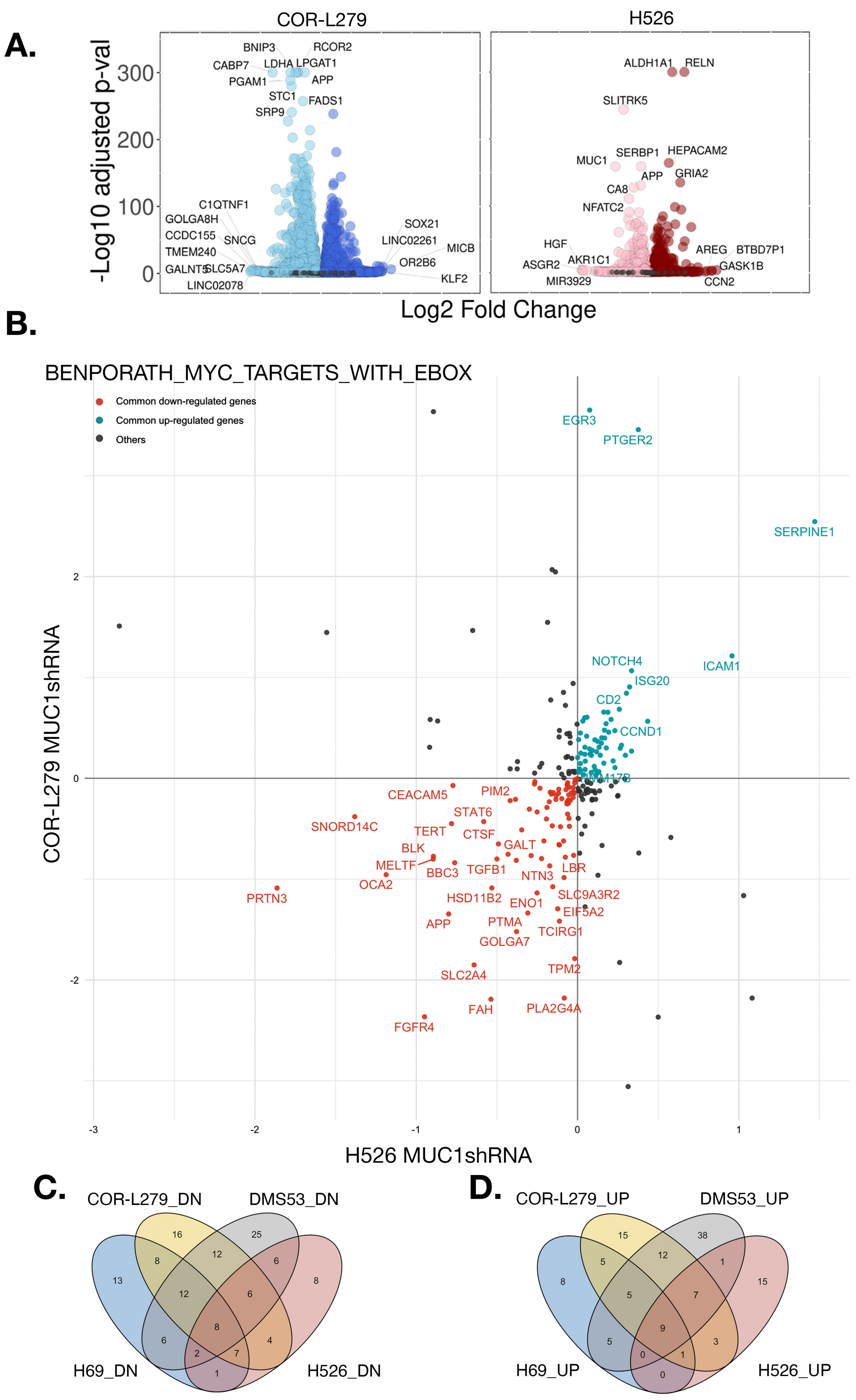
A.
COR-L279
H526
-Log10 adjusted p-val
300
200
100
0
BNIP3
RCOR2
CABP7
LDHA
LPGAT1
PGAM1
APP
STC1
FADS1
SRP9
C1QTNF1
GOLGA8H
CCDC155
SNCG
TMEM240
GALNT5
SLC5A7
LINC02078
SOX21
LINC02261
MICB
OR2B6
KLF2
ALDH1A1
RELN
SLITRK5
MUC1
SERBP1
HEPACAM2
APP
GRIA2
CA8
NFATC2
HGF
ASGR2
AKR1C1
MIR3929
AREG
BTBD7P1
GASK1B
CCN2
Log2 Fold Change
B.
BENPORATH_MYC_TARGETS_WITH_EBOX
Common down-regulated genes
Common up-regulated genes
Others
EGR3
PTGER2
SERPINE1
NOTCH4
ICAM1
ISG20
CD2
CCND1
CEACAM5
PIM2
STAT6
SNORD14C
TERT
CTSF
GALT
BLK
MELTF
BBC3
TGFB1
NTN3
LBR
OCA2
PRTN3
HSD11B2
ENO1
SLC9A3R2
EIF5A2
APP
PTMA
TCIRG1
GOLGA7
SLC2A4
TPM2
FAH
PLA2G4A
FGFR4
COR-L279 MUC1shRNA
2
0
-2
-3
-2
-1
0
1
H526 MUC1shRNA
C.
COR-L279_DN
DMS53_DN
H69_DN
H526_DN
16
25
8
12
6
13
12
6
8
8
6
4
2
7
1
D.
COR-L279_UP
DMS53_UP
H69_UP
H526_UP
15
38
5
12
1
8
5
7
15
9
5
3
0
1
0

Supplemental Figure S2. MUC1-C regulates common MYC target genes in H69, DMS53, COR-L279 and H526 cells. A. RNA-seq was performed in triplicate on COR-L279/tet-MUC1shRNA and H526/tet-MUC1shRNA cells treated with vehicle or DOX for 7 days. The datasets were analyzed for effects of MUC1-C silencing on repressed and activated genes as depicted by the Volcano plots. **B.** Overlap of MUC1-C-regulated BENPORATH MYC TARGETS WITH EBOX genes in COR-L279 and H526 cells. **C and D.** Common BENPORATH MYC TARGETS WITH EBOX genes downregulated (**C**) and upregulated (**D**) by MUC1-C silencing in the indicated SCLC cells.

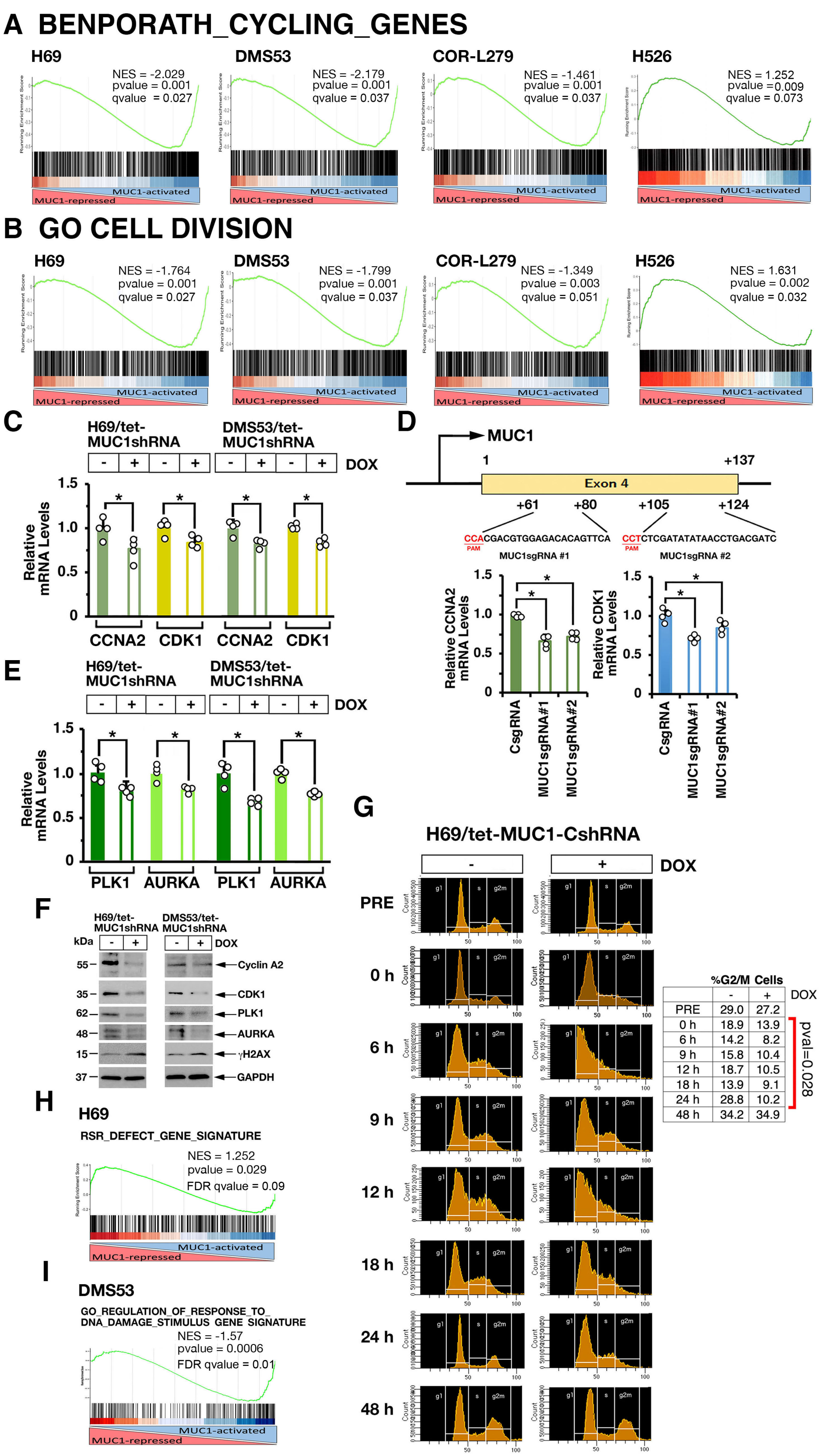

	-	+
PRE	29.0	27.2
0 h	18.9	13.9
6 h	14.2	8.2
9 h	15.8	10.4
12 h	18.7	10.5
18 h	13.9	9.1
24 h	28.8	10.2
48 h	34.2	34.9

Supplemental Figure S3. MUC1-C drives expression of MYC target and cell cycle genes in SCLC cells. A and B. H69, DMS53, COR-L279 and H526 RNA-seq datasets were analyzed with GSEA using the BENPORATH

CYCLING GENES (**A**) and GO CELL DIVISION (**B**) gene signatures. **C.** H69/tet-MUC1shRNA (left) and DMS53/tet-MUC1shRNA (right) cells treated with vehicle or DOX for 7 days were analyzed for the indicated mRNA levels by qRT-PCR. **D.** *MUC1 e*xon 4 encoding MUC1-C was targeted with the indicated MUC1sgRNAs (Supplemental Table S2). H69 cells transduced with CsgRNA, MUC1sgRNA#1 and MUC1sgRNA#2 were analyzed for the indicated mRNA levels by qRT-PCR. **E.** H69/tet-MUC1shRNA (left) and DMS53/tet-MUC1shRNA (right) cells treated with vehicle or DOX for 7 days were analyzed for the indicated mRNA levels by qRT-PCR. The results (mean±SD of four determinations) are expressed as relative mRNA levels compared to that obtained for vehicle-treated cells (assigned a value of 1). **F.** Lysates from H69/tet-MUC1shRNA (left) and DMS53/tet-MUC1shRNA (right) cells treated with vehicle or DOX for 7 days were immunoblotted with antibodies against the indicated proteins. **G.** H69/tet-MUC1shRNA cells were treated with vehicle or for 7 days (PRE) and then cultured in the (i) presence of 2 mM thymidine for 18 h, (ii) absence of thymidine for 9 h, and (iii) presence of 2 mM thymidine for an additional 18 h. Cells released from the double-thymidine block were harvested at the indicated times and monitored for cell cycle distribution by flow cytometry (left). Silencing MUC1-C was associated with a significant decrease in the percentage of cells in G2/M phase at 6 to 24 h after release (right). **H and I.** The indicated RNA-seq datasets were analyzed with GSEA using the RSR_DEFECT_GENE_SIGNATURE (**H**) and the GO_REGULATION_OF_RESPONSE_TO_DNA_DAMAGE_STIMULUS (**I**) gene signatures.

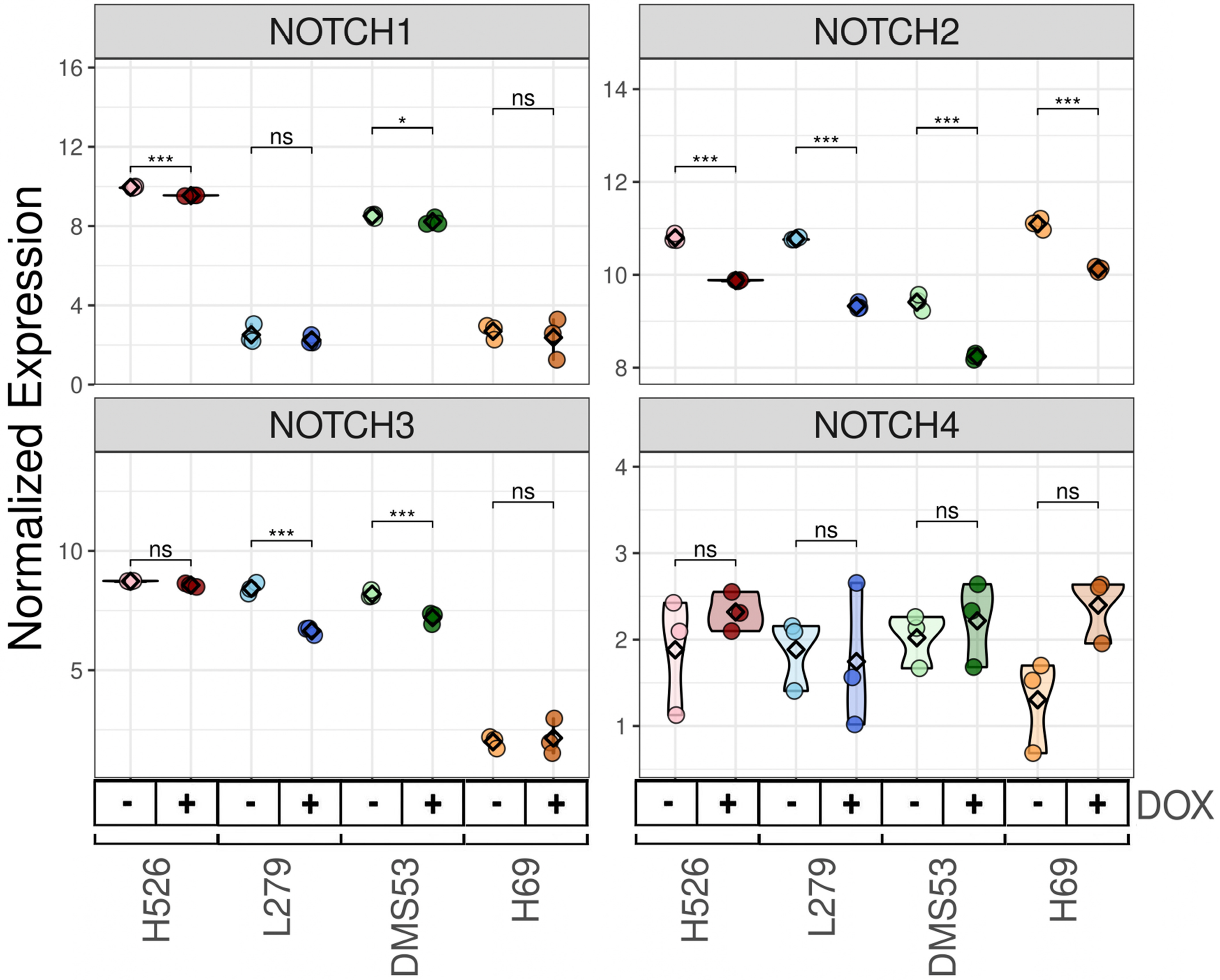

Supplemental Figure S4. Effects of MUC1-C silencing on NOTCH1-4 mRNA levels in the indicated SCLC cells without and with MUC1 silencing.

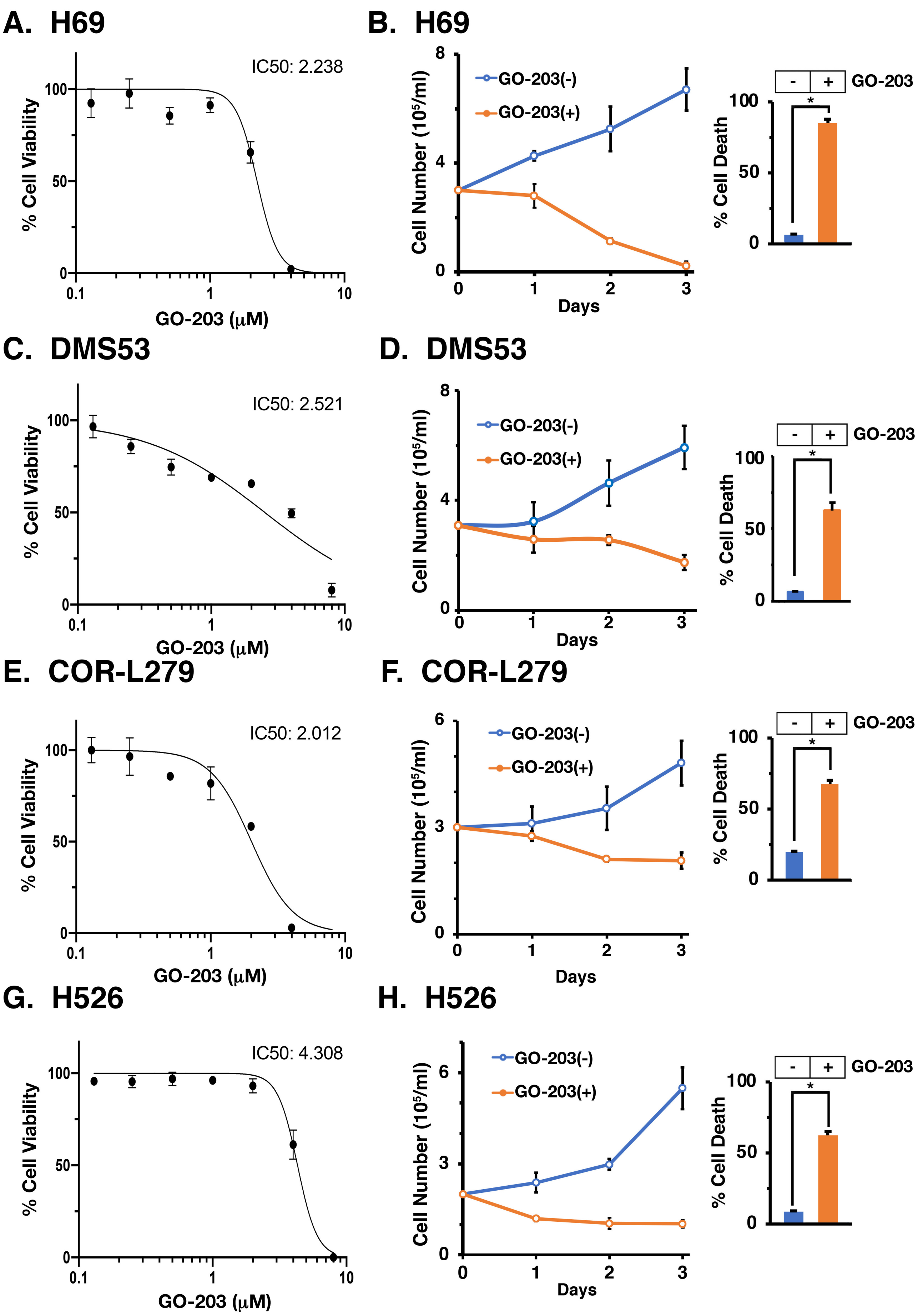
A. H69
IC50: 2.238
% Cell Viability
GO-203 (μM)
B. H69
GO-203(-)
GO-203(+)
Cell Number (10^5/ml)
Days
% Cell Death
GO-203
C. DMS53
IC50: 2.521
% Cell Viability
GO-203 (μM)
D. DMS53
GO-203(-)
GO-203(+)
Cell Number (10^5/ml)
Days
% Cell Death
GO-203
E. COR-L279
IC50: 2.012
% Cell Viability
GO-203 (μM)
F. COR-L279
GO-203(-)
GO-203(+)
Cell Number (10^5/ml)
Days
% Cell Death
GO-203
G. H526
IC50: 4.308
% Cell Viability
GO-203 (μM)
H. H526
GO-203(-)
GO-203(+)
Cell Number (10^5/ml)
Days
% Cell Death
GO-203

Supplemental Figure S5. Effects of targeting MUC1-C with GO-203 on SCLC cell proliferation and death. A-H. H69 (**A,B**), DMS53 (**C,D**), COR-L279 (**E,F**) and H526 (**G,H**) cells treated with (i) the indicated concentrations of GO-203 for 3 days were monitored for cell viability (**A,C,E,F)** and (ii) 5 μM GO-203 were monitored for effects on proliferation at the indicated times (**B,D,F,H;** left) and % cell death on day 3 (**B,D,F,H;** right). The results are expressed as the mean±SD of three determinations.

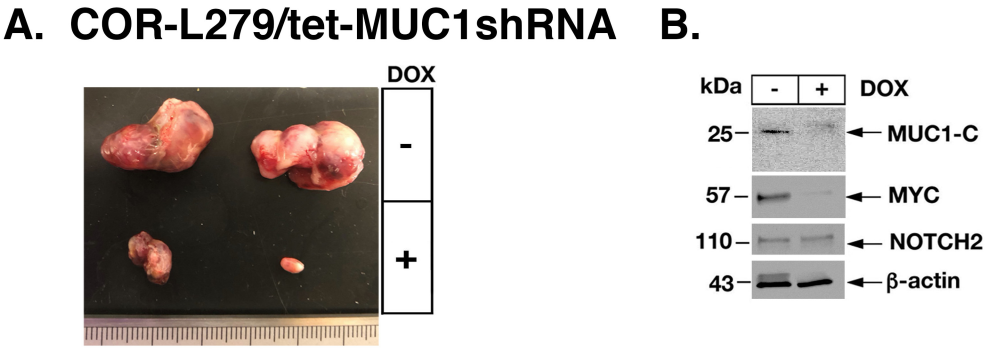

Supplemental Figure S6. MUC1-C is necessary for growth of COR-L279 tumors. A. Six-week old nude mice were injected subcutaneously in the flank with 3 × 10^6 COR-L279/tet-MUC1shRNA cells. Mice were pair-matched into two groups when tumors reached 100–150 mm^3 and were fed without and with DOX. Tumors harvested on day 13 are shown from control and DOX-treated mice. **B.** Lysates from control and DOX-treated tumors were immunoblotted with antibodies against the indicated proteins.

Table S1. Primers used for qRT-PCR analysis.

Primer	FWD	REV
**MUC1-C**	AGACGTCAGCGTGAGTGATG	GCCAAGGCAATGAGATAGAC
**CCNA2**	CGCTGGCGGTACTGAAGTC	GAGGAACGGTGACATGCTCAT
**CDK1**	TTTTCAGAGCTTTGGGCACT	CCATTTTGCCAGAAATTCGT
**PLK1**	CACCAGCACGTCGTAGGATTC	CCGTAGGTAGTATCGGGCCTC
**AURKA**	CCACCTTCGGCATCCTAATA	TCCAAGTGGTGCATATTCCA
**NOTCH2**	AAGAAACAGAGGATGACAGG	ACTGAACCTGACCGTACATGGTCTGAGTCTTGAACACA
**GAPDH**	CCATGGAGAAGGCTGGGG	CAAAGTTGTCATGGATGACC

Table S2. CsgRNA and MUC1sgRNA sequences

	**Top**	**Bottom**
**CsgRNA**	5′-caccgTGACCTCTGAGGAATTCACA-3′	5′-aaacTGTGAATTCCTCAGAGGTCAc-3′
**MUC1sgRNA#1**	5′-caccgGATCGTCAGGTTATATCGAG-3′	5′-aaacCTCGATATAACCTGACGATCc-3′
**MUC1sgRNA#2**	5′-caccgTGAACTGTGTCTCCACGTCG-3′	5′-aaacCGACGTGGAGACACAGTTCAc-3′
**NOTCH2sgRNA**	5′-caccgTTGATGTCCATCTCACAACG-3′	5′-aaacCGTTGTGAGATGGACATCAAc-3'

Table S3. Primers used for ChIP-PCR.

**NOTCH2-Promoter Region**	
Forward CCTCTAAGGGTGGGCATTTAT	Reverse GAGACCAGCTAAGCTAAGGATATT
**NOTCH2-Enhancer Region**	
Forward TTGCACACCCGAGAAAGT	Reverse TCCTGCTTCAAAGGCTCAG
